# Constrained distance transforms for spatial atlas registration

**DOI:** 10.1186/s12859-015-0504-5

**Published:** 2015-03-18

**Authors:** Bill Hill, Richard A Baldock

**Affiliations:** MRC Human Genetics Unit, MRC IGMM, University of Edinburgh, Edinburgh, EH4 2XU UK

**Keywords:** Constrained distance transform, Non-rigid registration, Atlas informatics

## Abstract

**Background:**

Spatial frameworks are used to capture organ or whole organism image data in biomedical research. The registration of large biomedical volumetric images is a complex and challenging task, but one that is required for spatially mapped biomedical atlas systems. In most biomedical applications the transforms required are non-rigid and may involve significant deformation relating to variation in pose, natural variation and mutation. Here we develop a new technique to establish such transformations for mapping data that cannot be achieved by existing approaches and that can be used interactively for expert editorial review.

**Results:**

This paper presents the *Constrained Distance Transform* (*CDT*), a novel method for interactive image registration. The CDT uses radial basis function transforms with distances constrained to geodesics within the domains of the objects being registered. A geodesic distance algorithm is discussed and evaluated. Examples of registration using the CDT are presented.

**Conclusion:**

The CDT method is shown to be capable of simultaneous registration and foreground segmentation even when very large deformations are required.

## Background

The use of spatially mapped databases has become widespread within the biomedical research community [1]. Many of these databases, such as EMAGE [2] and the Allen Brain Atlas [3], are based on volumetric atlases or reference models with assay data mapped onto the atlas models through non–linear spatial transformations or warps. Compared to the warps used in medical imaging to register images in a longitudinal study or between patients, the warps required for these atlases are complex and challenging. With the significant challenges including variations in pose, mutant phenotypes, inter–species registration and the frequently non-corresponding image values due to gene expression or other spatial signals. Yet it is often in these most challenging of cases that the biological interest is greatest.

There may be only a weak relationship between the image values of an assay and the appropriate atlas image. This may be because of differences in imaging modalities, but more critically because the assay image is of a spatially distributed signal and the presence of that signal obscures or modifies the structural image across modalities. In such cases it is not possible to acquire a reference image closely similar to the atlas model. When combined with extreme variation in pose, these problems may result in an algorithm struggling to find points of correspondence between such atlas and assay images; an expert however can often find these points relatively quickly. In such cases the time spent by an expert may be significantly less than that spent correcting correspondences found automatically by an algorithm.

Radial basis function (*RBF*) transforms are frequently used for interactive image registration and perform well for small deformations. However, when the deformation gradients are large these methods may produce non-diffeomorphic, mirrored or extremely distorted mappings [4]. Methods based on elasticity with a uniform homogeneous material are also unable to register images where large deformations are required because of pose, giving rise to severe image distortion. In many cases the problematic large deformation gradients required for these methods are necessary only because the methods do not respect object boundaries. Current methods for registering images where large deformations are required include articulated and fluid models. Articulated models have been used for registering hand radiographs [5], in which skeletons composed of articulated rods are registered using landmarks at the ends of the rods and the displacements away from the rods are interpolated using weighted combinations of affine transforms. Fluid models based on solving viscoelastic systems have been developed but the fluid deformation model, like the articulated model, is often inappropriate [4] and the computation time for fluid models may be prohibitive.

For an interactive registration method to be useful it must be possible to compare the registered assay image with the model in a reasonable time. RBF transforms have a high computational cost because they typically rely on the evaluation of transcendental or other expensive functions, with the number of evaluations being proportional to the image volume and number of landmarks. Large numbers of landmarks may be required to force acceptable deformations, together with 3D image volumes, these may make the computation time unacceptable for interactive use. The computational cost of basis function evaluations can however be reduced by using mesh based methods, with evaluation of the basis functions only at the nodes off the mesh and a low cost interpolation within the mesh elements [6,7].

**Figure 1:**
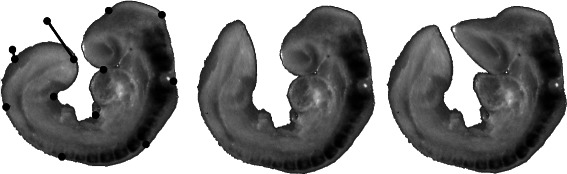
**Locality and deformation.** From left to right: Source image (from EMAGE) showing fixed points and displacements, source image transformed after applying a CDT and source image transformed after applying an unconstrained RBF transformation.

In this paper we describe a mesh based image registration method, particularly suited to interactive image registration and which is suitable for 2 and 3-dimensional images in which large deformations (for example those due to pose) are required. Our method uses RBFs, but with distances computed along geodesics that are within an object rather than Euclidean distances which may cross object boundaries. By using geodesic distances, points which are distant within an object have large distances between them irrespective or whether or not they are close in Euclidean space and object boundaries are respected; for example, in Figure 1 the head and tail are close in Euclidean space yet distant when geodesic distances are used. The RBF, computed using geodesic distances, results in a mesh based transform which is then used to warp either the source or target. We call this the *constrained distance transform* (*CDT*). Because our object representation has a direct relevance to spatial mapping, we describe it briefly. We discuss geodesic distance algorithms and present our mesh based fast marching algorithm. The advantages of mesh based transforms are discussed and our mesh based RBF transform implementation is described. These components are then drawn together in an overall description of the CDT method. We present experimental results for the geodesic distance algorithm and from the registration of challenging biological data.

## Method

### Object representation

Images are frequently represented as simple rectangular or cuboid arrays of image values, but there are many situations in which it is useful to separate the representations of regions of space or *domains* from the image values that the domains cover. Such representations allow multiple objects with different domains to share a single set of values or *value table*; alternatively they may allow multiple objects with different value tables to share a single domain. In many cases it may be only the domains (and not the values within them) that are of interest and in these cases operations on the domains may be made more efficient through an appropriate representation such as interval coding [8]. This separation of domain and values is particularly appropriate for spatial atlases, where spatial queries can then be more efficiently handled. Using this representation; an image object *O* will always have a domain *Ω* and may have a value table *V*. A transformation (*T*) may then be expressed as a mapping from a source domain to a target domain: *T*:*Ω*_*s*_→*Ω*_*t*_.

### Radial basis functions

Given a source object *O*_*s*_(**x**) with **x**∈*Ω*_*s*_ and image values *V*_*s*_(**x**); likewise a target object *O*_*t*_(**u**) with **u**∈*Ω*_*t*_, then the transformation *T*:*Ω*_*s*_→*Ω*_*t*_ will have displacements *Δ* **u**=**u**−**x**. If some subset of *landmarks* can be established, either by an expert or by some algorithm, then a number of methods exist for approximating or interpolating displacements throughout a domain from discrete landmarks. One such method is the Radial Basis Function (*RBF*) transform, in which all displacements are approximated or interpolated by the weighted sum of radially symmetric functions with the form:

(1) $$ \Delta \mathbf{u}_{j} = P_{j}(\mathbf{x}) + \sum\limits_{i = 1}^{i = N} {\lambda_{i,j} f(\|\mathbf{x} - \mathbf{x_{i}}\|)}.  $$

Here **u**_*j*_ is the *j*t́h component of **u**, *P* is a first order polynomial, *N* is the number of landmark points **x**_*i*_,*λ*_*i*_ are the basis function coefficients and *f* is the basis function. The polynomial and basis function coefficients are computed from the design equation

(2) $$ \left(\begin{array}{cc} O_{0} & X \\ X^{T} & R \end{array} \right) \left(\begin{array}{c} a \\ \lambda \end{array} \right) = \left(\begin{array}{c} O_{1} \\ D \end{array} \right),  $$

where *O*_0_ is a zero matrix, *O*_1_ is a zero column vector, *a* is a column vector of the polynomial coefficients, *λ* is a column vector of the basis function coefficients, *R* is a symmetric matrix with the radial basis function values evaluated at the landmarks, *X* is a matrix containing the coordinates of the landmarks and *D* is a column vector of the displacements for the landmarks. The design equation may then be solved using a linear system solver such as singular value decomposition [9], although in practise it is often beneficial to rescale the parameters to reduce the condition number of the design matrix [6].

A number of radial basis functions have been proposed for non–linear image registration [6,10-12]. These include the thin plate spline (*TPS*), the multiquadric (*MQ*), the inverse multiquadric (*IMQ*) and compactly supported RBFs such as those of Wendland [13]. The form of these RBFs is outlined in Table 1. Although the TPS appears to be frequently used for image registration [4,14], comparisons of the TPS with the MQ and other global RBFs have shown the MQ to have better stability and frequently better accuracy [15]. A possible reason for the observed stability of the MQ is the parameter *c*. The value chosen for this parameter is application specific and can be thought of as balancing the accuracy against the smoothness of the deformation. In practice we have chosen to set the parameter using *c*=*δ* *r*_*max*_, where *r*_*max*_ is chosen to be maximum extent of the source object, that is *r*_*max*_= max*i*=*x*,*y*[,*z*](*m* *a* *x*_*i*_−*m* *i* *n*_*i*_). We have observed the parameter *δ* to have a useful range [0.001-0.5].

**Table 1:** **Radial basis functions and their form**

**Radial basis function**	**Form**
Thin plate spline	*ϕ*(*r*)=*r* ^2^ log*r*
Multiquadric	$ \phi (r) = (r^{2} + c^{2})^{\frac {1}{2}}$
Inverse multiquadric	$\phi (r) = (r^{2} + c^{2})^{-\frac {1}{2}}$
Wendland’s functions	$\phi (r) = \left \{ \begin {array}{r@{\quad :\quad }l} p(r) & 0 \leq r \leq 1 \\ 0 & r > 1 \end {array} \right. $

Outline form of radial basis functions based on the thin plate spline, multiquadric, inverse multiquadric and Wendland’s functions. In Wendland’s functions *p(r)* is a univariate polynomial.

### Geodesic distances

RBFs conventionally use Euclidean distances, however in the SCDT we wish to constrain the transformations using distances evaluated along paths that are constrained to the object’s domain. The minimum distance between two points within a convex domain is always the Euclidean distance, but when the domain is non-convex and the path between the two points is constrained to the domain then the Euclidean distance is the lower limit for the *geodesic distance* [16]. Reviews and evaluations of geodesic distance transform algorithms, are given in [17] and [18]. It is this geodesic distance that is used in the CDT. One of the first algorithms for computing geodesic distances was that of Piper and Granum [19], which like many later algorithms is based on region growing.

An early implementation of the CDT used a region growing algorithm based on morphological operators (similar to [19]) to compute geodesic distances, but as this was computationally prohibitive for interactive landmark placement a faster mesh based algorithm was developed. Because for the CDT geodesic distances are only required at the nodes of the mesh and at the landmark points, this led us to develop an algorithm for computing the geodesic distances at only the mesh nodes directly within the mesh using a fast marching algorithm.

**Figure 2:**
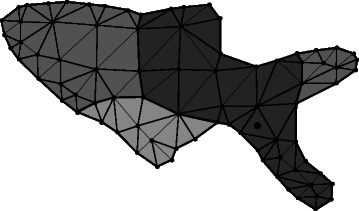
**Distance propagation (first stage).** Illustration of the nearest neighbour line of sight distance propagation algorithm. Distances are propagated from a seed vertex, here shown as a black circle within a triangular mesh element (*e*_0_ in the text). Elements with all nodes found to be within line of sight of the seed are shown with dark shading; elements with all nodes which are in line of sight of the seed but not found by the algorithm are shown with mid shading and elements with all nodes not in line of sight of the seed are shown with light shading.



A two stage algorithm for computing the geodesic distance of all nodes in a mesh from a seed vertex was developed. In the first stage a region is propagated out from the seed through those nodes that are within line of sight of the seed. During this initial propagation, distances at the nodes are computed using the Euclidean vector norm. In the second stage, the region is propagated further using a fast marching algorithm until the distance at all nodes is known. Both of these stages operate directly within the mesh. This two stage distance propagation algorithm has been implemented for both 2D and 3D meshes, but for simplicity only the 2D algorithm is described. The first stage uses a nearest neighbour line of sight algorithm (shown in Algorithm 1), in which the mesh element *e*_0_ containing the seed vertex is found first. An element queue is then initialised with the edge neighbours of the element *e*_0_. The queue is maintained so that the element removed is always that with the minimum distance between the node and the seed. Elements are removed from the queue until the queue is empty. For each element removed: If *e*_1_ removed from the queue has a node *n*_0_ which has not yet had it’s distance computed, then a test is made for whether this node is within line of sight of the seed node; if it is, then the distance for the node is computed and the edge neighbours of that element which still have nodes that have not had their distance computed are added to the queue. The test for *n*_0_ being within line of sight of the seed is purely local and consists of projecting a ray back from the *n*_0_ towards the seed. If the edge of *e*_1_ that is intersected by the ray has an element opposite to *e*_1_ and that element is known to be within line of sight of the seed, then *n*_0_ and hence *e*_1_ are classed as within line of sight of the seed. This algorithm is sufficient to initialise the second stage fast marching as it excludes all nodes that are not within line of sight of the seed, but it will not in general find all nodes that are within the line of sight. The result of the first stage in the distance propagation is illustrated in Figure 2, which clearly shows elements that have been incorrectly classified as not within line of sight of the seed. In the second stage a mesh based fast marching algorithm is used to propagate the known distance region throughout the remaining portion of the mesh. This closely follows the algorithms of Qian [20] which use causality preserving node orderings for the propagation. For obtuse elements which would violate causality (those for which the front would arrive at a distant node before a near one) Qian propagates the front to a node of a neighbouring element until one is found which does not make an obtuse angle. Because of the complication in implementing this, we have simply interpolated virtual nodes on the distance edges or faces of obtuse elements.

### Mesh based transforms

When a spatial transformation is applied to an object then it is common to apply a forward transformation to the object’s domain; image values within the domain are then interpolated in the space of the transformed domain using an inverse transformation. This two step transformation is in general necessary to avoid unassigned values in the transformed object. Many functions used for image transformation, such as radial basis functions, can not be analytically inverted. A mesh provides both a fast approximation to the RBF (or any other) displacement transform and allows the transformation to be inverted very simply and efficiently. Where the transform is costly to compute, then significant savings can be made by only computing the transform displacement at the nodes of a mesh. Wolberg [21] describes algorithms for 2D image re-sampling using regular quadrilateral meshes and scanline traversal. But there are significant advantages in using non-regular meshes: Biomedical images may contain little useful information in certain regions and these may have a courser mesh; the object being transformed may be non-rectangular and occupy a small part of it’s axis aligned bounding box or only the transformed domain may be required. All these factors allow significant performance increases to be achieved through the use of non–regular meshes, at the cost of increased complexity in the data structures and algorithms.

In this work the transformation of both object domains and values are accomplished using non-regular simplical conforming meshes. The use of conforming meshes simplifies image re-sampling in situations, such as limb articulation or lumen shrinkage, which would otherwise give rise to large mesh element deformations requiring complex re-meshing schemes. Objects are transformed by first defining a mesh covering their domain. This mesh then has displacements computed through the evaluation of the RBF nodal displacement with geodesic distances. The mesh is first used to forward transform the source object’s domain and then, if the source object has values associated with it, a new value table is created for the transformed domain and the new values interpolated using a mesh sweep line algorithm.

### Mesh generation

**Figure 3:**
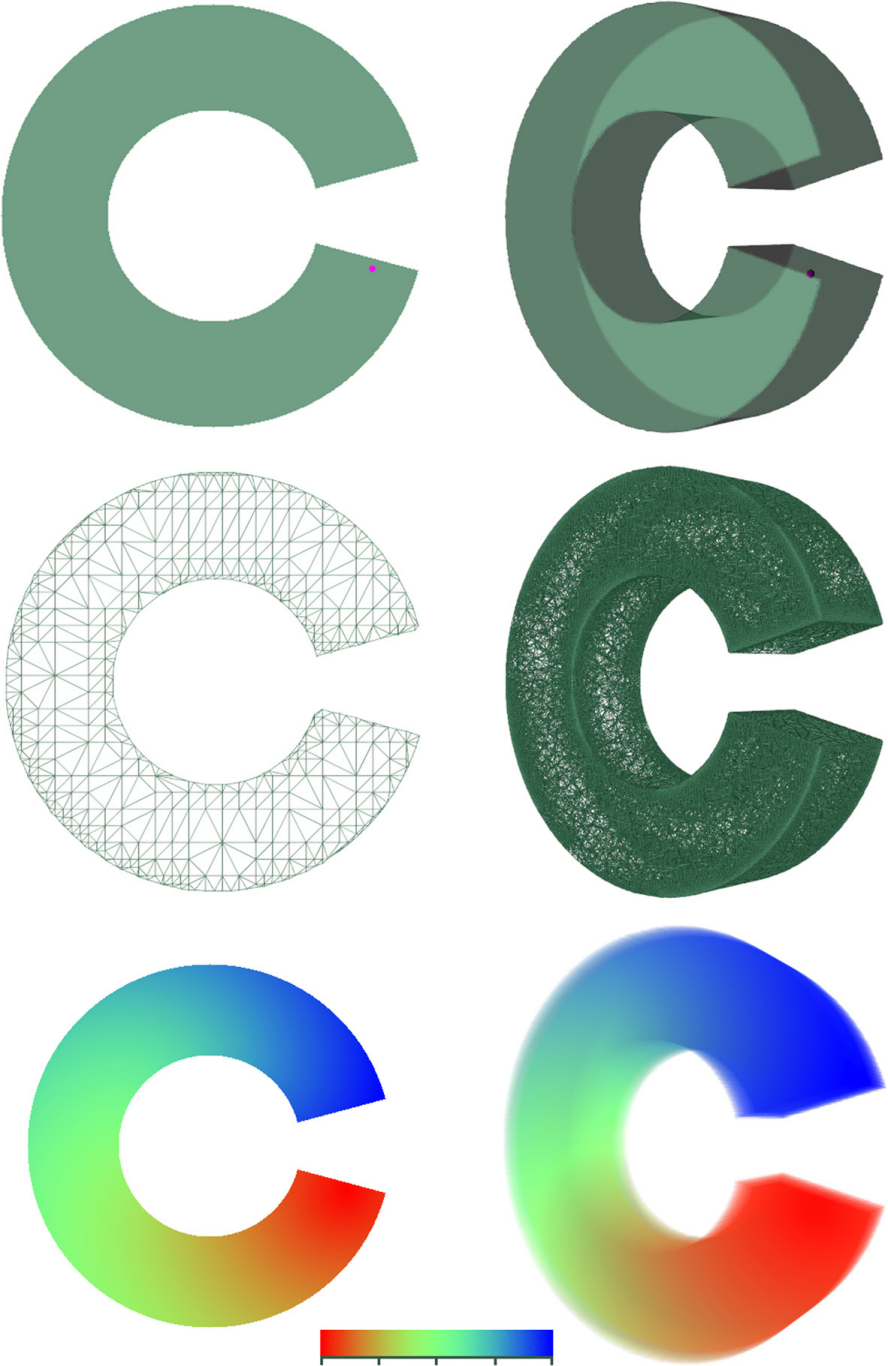
**Distance evaluation.** Left 2D and right 3D. From top to bottom: *C*2 and *C*3 domains showing seed point, *C*2_*m*_ (triangular) and *C*3_*m*_ (tetrahedral) meshes corresponding to the *C*2 and *C*3 domains, distance from the seed points evaluated within the domains shown using a linear rainbow scale with red minimum and blue the maximum distance (distance ranges for *C*2 and *C*3, 0-672 and 0-674 respectively).

**Figure 4:**
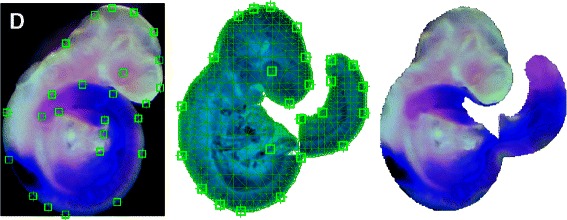
**2D one to many warp.** From left to right: Source image from EMAGE showing landmarks, target image with target mesh and landmarks, source image transformed and segmented using the CDT.

A simple mesh generator was implemented within Woolz to generate 2D and 3D meshes from object’s domains. This mesh generator, which is based on that of Zhang [22], decomposes the domain of an object into a balanced binary tree. The nodes of the tree are then used to form tiles of simplical mesh elements, with the elements of each tile determined through a simple classification of the binary tree node’s neighbour connectivity. Once the mesh is generated, a later step adjusts the position of mesh nodes which are outside of the object’s domain to fall within some minimum distance from it. While this approach produces valid conforming simplical meshes, the mesh quality is often poor, particularly so at the boundaries of 3D meshes. Because a mesh need only be generated once for each atlas model, it has proved practical to use an external high quality mesh generator. The 2D meshes used in this paper were generated within Woolz using the tiling algorithm described above, but all the 3D meshes were generated using Netgen [23]. Examples of these meshes can be seen in Figures 3 and 4. Irrespective of the method used, in all cases the mesh was generated so that it conformed to the atlas model’s domain. It would be possible to further refine the meshes varying the element size with image content, by for instance subdividing elements which correspond to regions of high image variance, but this has not yet been implemented.

### Constrained distance transforms

We have developed a registration method which we have called the *Constrained Distance Transform* (CDT). In this method displacements are computed within a mesh conforming to the target (or a source) domain by evaluating RBFs at the mesh nodes and using distances evaluated along paths constrained to geodesics within the mesh. Displacements within mesh elements are computed by interpolating nodal values. Using a CDT, connectivity and distance are defined by the domain conforming mesh and the problems associated with large deformations (such as to correct for pose) are significantly reduced. Regions close in Euclidean space may be easily pulled apart without distortion provided that they are distant with respect to geodesics in the conforming mesh. The CDT also allows multiple geodesically distant regions of a object to be fused.

### Implementation

The algorithms and data structures described in this paper have been implemented in C within the Woolz open source image processing system. Woolz also includes a Java Native Interface (JNI) binding making it possible to use Woolz from Java as well as native applications (https://github.com/ma-tech).

## Results and discussion

### Applicability to atlas registration

Because CDTs are invertible the mesh may be defined either on the source or target. This is important because for atlas systems, in which the target is an atlas model and the source an assay object, the number of meshes required is reduced to the number of atlas models and the additional cost of building these meshes may not be significant when compared to the total cost of building the atlas model. By defining the mesh using the atlas model, assay images are segmented from their background through their registration to the pre-segmented assay model, since values that fall outside of the mesh (and consequently the atlas model’s domain) are not mapped. We do not claim this segmentation to be novel, however it is an intrinsic feature of our method. In most cases this is desirable, but if ignored, it could lead to incorrect conclusions being drawn from the absence of mapped source regions.

### Distance evaluation errors

Simple 2D and 3D non-convex test domains *C*2 and *C*3 were created along with corresponding conforming meshes *C*2_*m*_ and *C*3_*m*_ as shown in Figure 3. These domains were created as they are sufficiently non-convex for a reasonable test case, while allowing simple closed form analytic expressions to be written for the exact constrained distance between any two vertices within them. Distances were then computed from a seed position within each of the two domains using exact analytic, morphological region growing and the mesh based algorithm described above. The mesh based algorithm was used both with and without the line of sight initialisation. The relative errors for the various algorithms with respect to the analytic solutions were then computed for the distance at the mesh nodes. Tables 2 and 3 display the percentage errors and execution times. These results show that in 2D the percentage mean error was between 60 and 140 times less for the mesh based algorithm than the morphological algorithms and that the line of sight initialisation reduced the percentage mean error by a factor of 14. In 3D, the percentage mean error for the mesh based algorithm was between 11 and 28 times lower; with the reduction through line of sight initialisation a factor of 3. The execution time for the mesh based algorithm was between a factor of 26 and 18 lower that the morphological algorithms for 2D and between a factor of 6 and 8 times lower for 3D. The line of sight initialisation had no significant effect on the execution times for either 2 or 3D. In the context of the CDT, constrained distances are only needed at mesh nodes and these are the only distances computed by the mesh based algorithm, in other applications interpolation might be needed to compute distances at all locations within a domain.

**Table 2:** **Errors and execution times for 2D distance evaluations**

**Algorithm**	**mesh**	**mesh_nlsi**	**c4**	**c8**	**oct**
% error	-0.0005 ±	-0.007 ±	-0.07 ±	-0.07 ±	-0.03 ±
	0.0007	0.009	0.02	0.02	0.01
Time (ms)	0.9	0.9	23	16	17

The Percentage error and execution time for 2D distance evaluations within the ***C2*** domain and ***C2***_***m***_ mesh; using the mesh based fast marching algorithm as described (mesh), the mesh based fast marching algorithm without line of sight initialisation (mesh_nlsi) and morphological region growing algorithms with 4 (c4), 8 (c8) and octagonal (oct) connectivity. The errors are shown as the percentage error in comparison to the analytic distances.

**Table 3:** **Errors and execution times for 3D distance evaluations**

**Algorithm**	**mesh**	**mesh_nlsi**	**c6**	**c26**	**oct**
% error	-0.004 ±	-0.013 ±	-0.07 ±	-0.11 ±	-0.045 ±
	0.002	0.006	0.02	0.02	0.008
Time (ms)	491	492	3832	2991	2878

The Percentage error and execution time for 3D distance evaluations within the ***C3*** domain and ***C3***_***m***_ mesh; using the mesh based fast marching algorithm as described (mesh), the mesh based fast marching algorithm without line of sight initialisation (mesh_nlsi) and morphological region growing algorithms with 6 (c6), 26 (c26) and octagonal (oct) connectivity. The errors are shown as the percentage error in comparison to the analytic distances.

### Displacement errors

Using a mesh in which the displacements are computed at it’s nodes and approximated within it’s elements results in some displacement error. These errors were evaluated for 3D conforming meshes with varying numbers of nodes but corresponding to the same domain.

#### Mesh resolution

To assess the impact of mesh resolution on the accuracy of the conforming mesh based approximation of the basis function displacements, meshes were constructed for a segmented embryo of the e-Mouse Atlas Project (EMAP) at various resolutions. Because it is likely that the displacement errors will be greatest at the centroids of the mesh elements, these displacements (as approximated by linear interpolation from the nodes), were compared with those computed by the RBF directly, using the normalised length of the error vector. The results shown in Table 4 display an approximate linear relationship between the cube root of the number of nodes in a mesh and the length of the error vector.

**Table 4:** **3D Displacement errors**

***N***	${\frac {1}{\sqrt [3]{N}}}$	***l***_***RBF***_	***l***_***MESH***_	***ε***
7963	0.050	65.7 ±78.5	65.4 ±78.1	0.0134 ±0.0268
13899	0.042	61.1 ±75.0	60.9 ±74.5	0.0095 ±0.0235
40255	0.029	62.2 ±75.4	62.0 ±74.5	0.0061 ±0.0278
117537	0.020	58.9 ±75.0	58.8 ±75.0	0.0046 ±0.0325
1294844	0.009	67.1 ±84.2	67.0 ±82.9	0.0023 ±0.0259

Variation in the length of the mesh element centroid displacement error vector with the number of nodes in the mesh; where *N* is the number of nodes in the mesh, *l*_*RBF*_ is the length of the RBF displacement vector, ***l***_***MESH***_ is the length of the interpolated displacement vector and ***ε=(l***_***RBF***_ ***−l***_***MESH***_ ***)/l***_***RBF***_. There is an approximate linear relationship between the displacement error (*ε*) and $\boldsymbol {\frac {1}{\sqrt [3]{N}}}$, with a correlation coefficient of 0.98.

#### Mesh defined locality

Within a CDT displacements are determined by the choice of RBF, landmarks and the constrained distance from the landmarks. A 2D assay image was selected from the e-Mouse Atlas Gene Expression database (EMAGE) in which the head and tail are close in Euclidean space (http://www.emouseatlas.org/emap/home.html and http://www.emouseatlas.org/emage/home.php). The image was segmented from its background and a conforming mesh was created for the segmented domain. Landmark points were then evenly distributed around the domain, with all the landmarks having zero displacements except for those at the tip of the tail which had a displacement set away from the head. Standard unconstrained RBF and CDT warps were applied using a MQ RBF in both warps. Figure 1 shows this image together with the landmark points and the displacement applied. The figure also shows the results of applying the CDT and an unconstrained RBF transform to the image. In the CDT warped image the head does not show any significant deformation despite the large deformation experienced by the tail, unlike the unconstrained RBF transform which resulted in large deformations to both the head and the tail. In both cases the same landmarks and displacements were used. The resulting warp shows how locality in CDTs is defined by the mesh.

### 2D Atlas registration

A 2D assay image was selected from the EMAGE database along with the corresponding 2D atlas image. The 2D atlas image is a projection of the 3D atlas model in a standard pose with the tail articulated away from the body to allow independent 2D spatial mapping on both parts. Using conventional mapping techniques this mapping would need to be performed separately for the body and tail, but since connectivity is defined by the mesh for CDT warps, they can be transformed simultaneously when using a mesh defined on the target. A mesh was constructed for the pre–segmented atlas (target) image and 25 landmark pairs were defined between the assay (source) and target images. Together with an inverse multiquadric RBF and *δ* value of 0.05 these defined the transformation from the assay (source) image to the atlas (target) image. Using a 2D CDT the assay image was registered to the atlas image and segmented in a single operation with the assay tail correctly mapped to the articulated tail. Image values in regions of the tail appear twice in the warped image illustrating that the CDT may produce one-to-many mappings. The source, target and transformed images can be seen in Figure 4.

**Figure 5:**
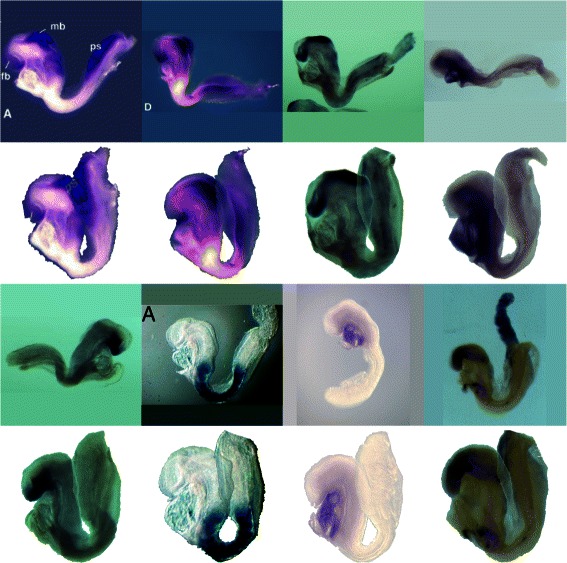
**2D assay warp.** Selected images from an EMAGE assay warped using the CDT; the alternate rows show the assay and warped assay images.

A set of 2D whole-mount assay images were selected from the EMAGE database for their extreme variability in pose. These were then warped to the appropriate atlas projection image using 2D CDTs. Figure 5 shows the wide variation in pose before warping and the uniformity after. As before the assay images were segmented to the domain of the target through the CDT warp. This figure also illustrates the ability of the CDT to produce many-to-one mappings.

### 3D Atlas registration

**Figure 6:**
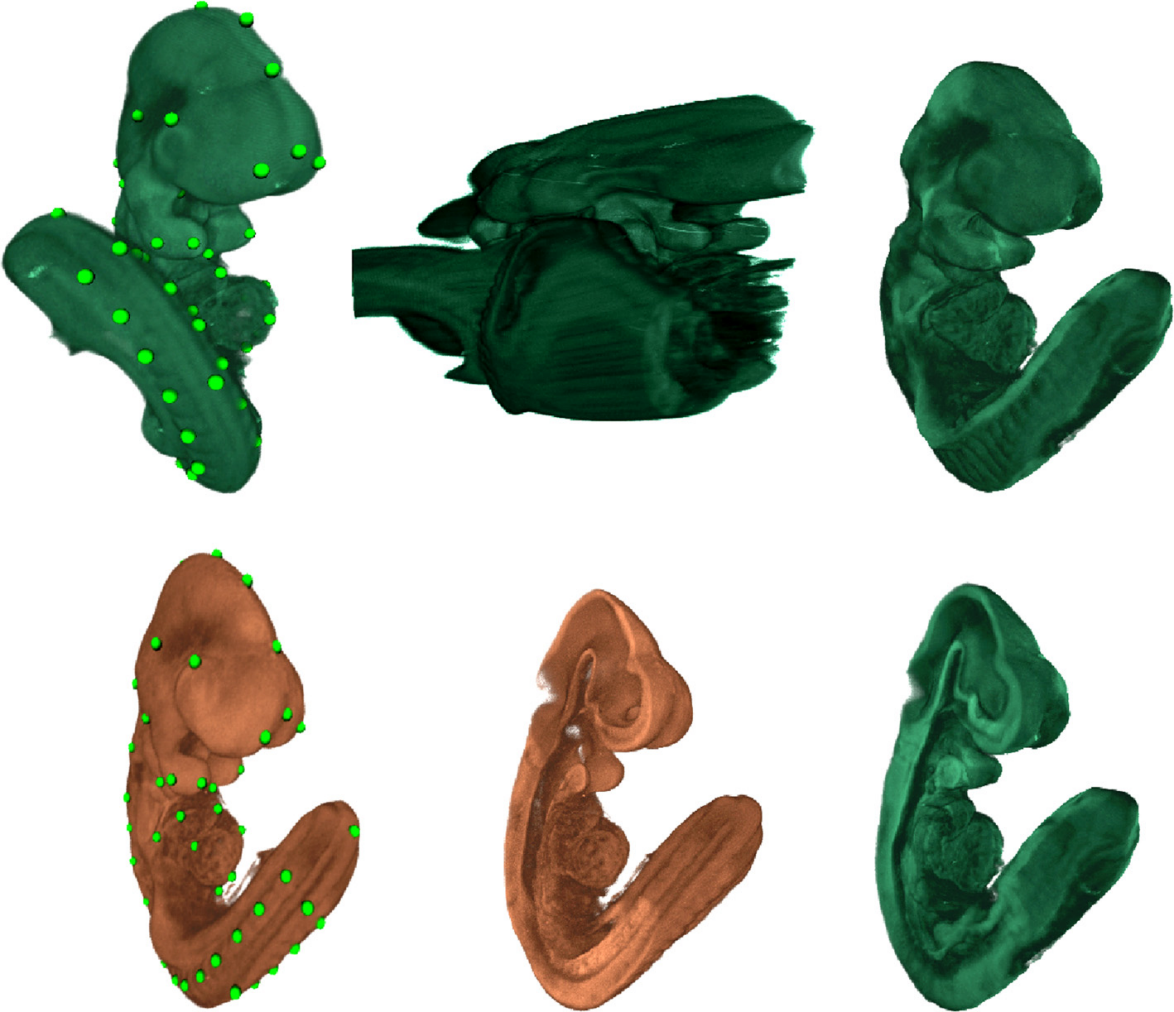
**3D warp with tail flip.** Clockwise from bottom left: Target volume, source volume, source warped to target using conventional RBF (truncated and rescaled), source warped to target using CDT, cut section through source warped using CDT and cut section through target. Landmarks are shown as spheres on the source and target volumes.

Two 3D (voxel) assay embryo images were selected from the EMAGE database, one with it’s tail curled to the left and the other with it’s tail curled to the right when viewed from the front. A foreground domain was segmented from the target image using simple grey value thresholding with some manual segmentation to ensure that there were no connecting bridges between the tail and the rest of the embryo. A conforming mesh was then generated and tie points (75 pairs) were defined interactively between points of correspondence on the volume rendered surface of the embryos. No internal correspondences were used. An inverse multiquadric RBF and *δ* value of 0.05 was used to define the transformation. These images are shown in Figure 6. Producing a warp between these images is extremely challenging for existing methods and the warp produced by a conventional RBF is unusable. Computing the CDT mesh displacements from the landmarks and warping this 3D image took 0.36 and 2.03 seconds respectively on a 3.4GHz Intel i7-2600 CPU. The displacement and warp evaluation times are shown in Table 5 to vary in proportion to the number of mesh nodes and it’s square root respectively for meshes with of the order of 10^4^ to 10^6^ nodes. When landmarks are edited interactively only the geodesic distances for the changed landmarks need to be recomputed, this can result in a significant saving over the total evaluation time when there are many landmarks.

**Table 5:** **3D Displacement and warp computation times**

***N***	***t***_***disp***_ ** (ms)**	***t***_***warp***_ ** (ms)**
7963	208	1764
13899	358	2027
40255	1336	2630
117537	4636	3678
1294844	65518	10749

The time taken to compute the mesh displacements and warp the 3D image using meshes of varying resolution; where *N* is the number of nodes in the mesh, *tdisp* and ***t***_***warp***_ are the distance and warp evaluation times. The displacement evaluation time is proportional to the number of nodes (correlation coefficient 0.9999), whereas the warp evaluation time is proportional to the square root of the number of nodes (correlation coefficient 0.9996).

## Conclusions

In this paper we have described a novel image registration algorithm, the CDT and demonstrated it’s applicability for atlas registration though it’s ability to register both 2D and 3D images to an atlas whilst performing simultaneous foreground segmentation in the presence of large deformations such as those required to correct for pose. We are unaware of any other published method which is capable of performing such registration.

A novel initialisation for computing distance within meshes using fast marching has been described which improves accuracy yet has minimal impact on the computation time.

The cost and difficulty of defining large number of landmarks required for very accurate alignment may be prohibitive and a two part approach may be preferred in which large deformations are computed using CDT and remaining small deformations through other algorithms.
